# Supplementary material for: Parental knowledge of early childhood caries and its association with bottle-feeding practices: A cross-sectional analysis in the Riyadh Province, Saudi Arabia
Source: PLoS One. 2026 May 13;21(5):e0348946. doi: 10.1371/journal.pone.0348946 (PMC13170824; doi:10.1371/journal.pone.0348946)
Supplement: Attachment — Submitted filename: Response_to_Reviewers.docx [file pone.0348946.s003.docx]

**Date:** March 18, 2026

**To:** Editorial Office, PLOS ONE

**Re:** Manuscript ID: PONE-D-26-02152

*Parental Knowledge of Early Childhood Caries and Its Association with Bottle-Feeding Practices: A Cross-Sectional Analysis in the Riyadh Province, Saudi Arabia*

Dear Editor and Reviewers,

We sincerely thank the Academic Editor and the reviewers for their thorough and constructive evaluation of our manuscript. We have carefully addressed each point raised, and the specific changes made are described in detail below. All modifications are highlighted in the Revised Manuscript with Track Changes.

**JOURNAL REQUIREMENTS**

*Reviewer comment: Please ensure that your manuscript meets PLOS ONE's style requirements, including those for file naming.*

**Response:** We have carefully reviewed the PLOS ONE formatting guidelines. The revised manuscript has been reformatted to comply fully with the PLOS ONE style templates. All section headers, in-text citations, and reference formatting have been verified.

*Reviewer comment: PLOS only allows data to be available upon request if there are legal or ethical restrictions on sharing data publicly.*

**Response:** We acknowledge this requirement. Upon reflection, we have determined that the dataset contains no personally identifiable information — all responses are categorical (age group, gender, income bracket, yes/no answers) and participant submission timestamps have been removed. The data are therefore suitable for full public sharing. The complete de-identified dataset is now provided as Supporting Information (S1 Data) accompanying this submission, in accordance with the IRB approval conditions of King Saud University Medical City (IRB Approval No. E-25-9545). The Data Availability Statement has been updated accordingly.

*Reviewer comment: If the reviewer comments include a recommendation to cite specific previously published works, please review and evaluate these publications.*

**Response:** We have reviewed all references recommended or implied by the reviewers. Relevant studies have been incorporated into the revised manuscript where appropriate. No reviewer explicitly mandated specific citations, but we have substantially expanded our reference list in response to the reviewers' general concerns about the breadth of the literature review (see Reviewer 1, Discussion comments below).

**REVIEWER #1 COMMENTS**

**Abstract**

*Reviewer comment: The abstract is clear and adequately reflects the study objectives, methods, and main findings. No major concerns.*

**Response:** We thank the reviewer for this positive assessment. No changes were made to the abstract.

**Introduction**

*Reviewer comment: The statement 'Early childhood caries (ECC) remains one of the most prevalent chronic diseases affecting infants and preschool children worldwide' should be supported by an appropriate reference.*

**Response:** A supporting reference has been added to this statement in the revised Introduction.

*Reviewer comment: The introduction is relatively lengthy and could be condensed.*

**Response:** The Introduction has been revised and condensed. Redundant sentences and passages that overlapped with the Discussion have been removed. The revised Introduction more concisely presents the epidemiological background, risk factors, the specific knowledge gap, and the study rationale.

**Materials and Methods**

*Reviewer comment: The sections 'Study Design', 'Study Setting and Duration', and 'Questionnaire Development and Content' contain overlapping information and would benefit from merging.*

**Response:** The content has been reorganized to eliminate redundancy and improve logical flow.

*Reviewer comment: The description of the questionnaire lacks sufficient detail for reproducibility. The authors should clearly specify: the total number of sections; the total number of questions; the number of questions within each section; the response format; and the scoring system.*

**Response:** We thank the reviewer for this important point. The Methods section now explicitly states that the questionnaire comprised 26 items across three sections: (1) Demographic Information (8 items); (2) Parental Knowledge of ECC (10 items); and (3) Parental Behavior regarding oral health and bottle-feeding practices (8 items). Responses were recorded using binary or multiple-choice formats. The full questionnaire is provided as Supplementary File S1.

*Reviewer comment: The eligibility criteria state that parents of children aged 1–5 years were included. However, the study objective and several sections refer to children under six years. This discrepancy should be clarified.*

**Response:** This inconsistency has been corrected throughout the manuscript. The eligible age range for children is 1–5 years (up to but not including the sixth birthday), consistent with the World Health Organization definition of early childhood caries for children under six years of age. We have standardized this phrasing to '1 to 5 years of age (under six years)' at first mention and '1–5 years' consistently thereafter.

**Results**

*Reviewer comment: The household income category 5,000–10,000 SAR should be presented with an approximate equivalent in USD or EUR.*

**Response:** We have added the approximate USD equivalent in parentheses in the income categories of Table 3 and in the relevant Results paragraph. At the time of data collection, 5,000–10,000 SAR corresponded to approximately USD 1,333–2,667.

*Reviewer comment: The notation 'N = 465' in table titles should use lowercase ('n'), as per standard scientific reporting.*

**Response:** Corrected. All instances of 'N =' in table titles and legends have been changed to 'n =' throughout the manuscript.

*Reviewer comment: Figure 1 is blurred and of low resolution. Figure 2 is also unclear and lacks sufficient visual quality. Both figures should be replaced with high-resolution versions.*

**Response:** Both figures have been replaced with high-resolution versions meeting PLOS ONE's minimum requirement of 300 DPI for half-tone figures and 600 DPI for line art. The figures were regenerated from the original data using dedicated graphical software. We have also verified that all text within the figures is legible at print size.

**Discussion**

*Reviewer comment: The abbreviation 'early childhood caries (ECC)' is repeatedly written in full after its first definition. The abbreviation alone should be used consistently after its first definition.*

**Response:** The full term 'early childhood caries (ECC)' is now defined at its first occurrence in the abstract and once more at its first occurrence in the main text; the abbreviation 'ECC' is used exclusively throughout the remainder of the manuscript.

*Reviewer comment: The discussion relies heavily on comparisons with studies from Saudi Arabia or neighboring regions. The authors should broaden the contextualization by including evidence from Europe, Asia, Africa, or the Americas.*

**Response:** We have substantially expanded the Discussion to incorporate evidence from diverse international settings. This broadened perspective enhances the international relevance of our findings and allows readers to contextualize our results within the global epidemiology of ECC.

*Reviewer comment: The manuscript currently includes a limited number of recent and high-impact references. The reference list should be expanded and include at least ~30 recent references, particularly international studies on parental knowledge and ECC.*

**Response:** The reference list has been substantially expanded. The revised manuscript now includes 37 references in total, of which the majority were published within the past ten years. We have prioritized high-impact peer-reviewed studies on parental knowledge, attitudes, and practices (KAP) related to ECC, including international systematic reviews and meta-analyses where available.

**Conclusions**

*Reviewer comment: The conclusions are consistent with the results and appropriately formulated.*

**Response:** We thank the reviewer for this affirmation. The Conclusions section has been lightly revised to incorporate the specific recommendations raised by Reviewers 2 and 3, including actionable public health recommendations and suggestions for future longitudinal research.

**REVIEWER #2 COMMENTS**

*Reviewer comment: Overall, minor English proofreading is needed to improve clarity and flow.*

**Response:** The entire manuscript has undergone thorough English language editing. Sentence structure, grammar, and lexical clarity have been reviewed and improved throughout. We paid particular attention to the Methods and Discussion sections, which were identified as areas requiring the most improvement. A professional language editing service was used in addition to author-level revision.

*Reviewer comment: Keywords could be refined to enhance indexing and search visibility.*

**Response:** The keywords have been revised. The updated keyword list is: early childhood caries; parental knowledge; bottle-feeding; oral health; cross-sectional study; Saudi Arabia; pediatric dentistry; health behavior. These terms have been selected to improve discoverability across major biomedical databases including PubMed and Scopus.

*Reviewer comment: Kindly explain the questionnaire validation process (1–2 sentences).*

**Response:** A brief description of the validation process has been added to the Methods section. Content validity was established by a panel of five expert dentists who assessed the relevance and clarity of each item. A pilot study involving 40 participants was conducted to assess comprehensibility; as no substantial modifications were required, the pilot data were retained and included in the final analysis. Content validity indices (CVI and CVR) are reported in response to Reviewer 3 below.

*Reviewer comment: Rewrite the conclusion section. I suggest adding more specific actionable recommendations.*

**Response:** The Conclusions section has been rewritten to include clear, specific, and actionable recommendations. These now include: (1) integration of ECC prevention counseling into routine well-child visit protocols within the Saudi primary healthcare system; (2) development of culturally adapted Arabic-language educational materials targeting parents of young children; (3) training programs for primary healthcare providers on anticipatory guidance for oral health; and (4) policy-level recommendations for the inclusion of structured dental health education in the national maternal and child health framework.

**REVIEWER #3 COMMENTS**

**Methods**

*Reviewer comment: What was the sampling technique? Was it a convenience sample? Please mention clearly.*

**Response:** We thank the reviewer for this important point. The sampling technique has now been explicitly described in the Methods section. The study employed a convenience sampling approach, whereby parents of pediatric patients attending the Dental Hospital, King Saud University Medical City (KSUMC), Riyadh, during the study period were approached and invited to participate. This has been acknowledged as a limitation of the study in the revised Limitations section.

*Reviewer comment: The validity wasn't mentioned. Please state clearly the values of the CVI and CVR.*

**Response:** We apologize for this omission. The Methods section now reports that content validity was assessed by a panel of five experts. The Content Validity Index (CVI) for the overall questionnaire was 0.89 (item-level CVI range: 0.81–0.92), and the Content Validity Ratio (CVR) was 0.91, both indicating adequate content validity. These values have been added to the Questionnaire Validation subsection.

*Reviewer comment: Add a sentence explaining the scoring system of the questionnaire for knowledge or behavior.*

**Response:** A sentence has been added to the Methods section describing the scoring system: ‘Each correct response to a knowledge item was assigned a score of 1 and each incorrect or ‘I don’t know’ response was assigned a score of 0. Higher scores indicate greater parental knowledge of ECC.’

*Reviewer comment: In the statistical analysis: Chi-square tests or Fisher's exact tests were mentioned, but in the table an independent t-test was calculated instead. Please correct.*

**Response:** We thank the reviewer for identifying this inconsistency. The statistical methods reported in the text and those applied in the analysis have been reconciled. The Methods section now accurately states that independent samples t-tests were used to compare mean knowledge scores between two-category groups, while chi-square tests (or Fisher's exact tests where expected cell counts were below 5) were used to assess associations between categorical variables. The relevant table headings and footnotes have been corrected accordingly.

**Results**

*Reviewer comment: You conduct 'multivariable linear regression,' which is suitable for continuous outcomes, while in Table 1 you only present knowledge as number and percentage. Recommend adding a row illustrating the total knowledge score.*

**Response:** A row has been added to Table 1 presenting the total knowledge score as a continuous variable (mean ± SD), facilitating the use of multivariable linear regression as the primary inferential method. This addition clarifies the analytical rationale and makes the data presentation internally consistent.

*Reviewer comment: In the table where you represent the mean score, you should present Standard Deviation (SD) as (mean ± SD).*

**Response:** All mean values presented in the tables have been reformatted to 'mean ± SD' as per standard scientific convention. This correction has been applied consistently across all relevant tables.

*Reviewer comment: Since it is a cross-sectional study, the term 'predictors' for knowledge is not appropriate. Replace it with 'factors associated.'*

**Response:** The term 'predictors' has been replaced with 'factors associated with' throughout the Results and Discussion sections, as this language is more appropriate for the cross-sectional design of the study. This limitation has also been explicitly acknowledged in the Limitations section: 'As a cross-sectional study, causal inference cannot be established, and regression coefficients should be interpreted as measures of association rather than prediction.'

**Discussion**

*Reviewer comment: Recommend mentioning Health Literacy vs. Health Knowledge. Add a sentence discussing how 'Health Literacy' (the ability to apply information) might be the missing link.*

**Response:** An important paragraph has been added to the Discussion addressing the distinction between health knowledge and health literacy. We now note that despite moderate levels of parental knowledge, high-risk feeding behaviors persisted in our sample, suggesting that knowledge alone is insufficient to drive behavioral change. We introduce the concept of health literacy as a potential mediating factor, acknowledging that cultural norms, convenience, and infant-soothing needs may override biological knowledge of caries risk. We recommend that future interventions target health literacy and behavioral activation in addition to knowledge transfer.

*Reviewer comment: 60.9% of children had received dental treatment — since the children are aged 1–5, this is exceptionally high. Please justify this point.*

**Response:** This point is well taken. In the revised Discussion, we explicitly address this finding. Studies have reported that ECC is an economic burden on families and society as the treatment requires intensive dental care (7, 10, 23, 28, 29, 30). However, as primary teeth exfoliate, ECC was not considered an emergency dental disease. As studies have been carried out on the prevalence of caries among children above 12 years, there is a scarcity of literature regarding ECC among children below 12 years (31, 32, 33). Studies have reported that ECC affects around 49% of preschoolers globally (34, 35). Moreover, the distribution of ECC varies according to geographic area. A systematic review reported that higher prevalence of ECC has been reported in Asian and European countries (20). Studies reporting the global burden of dental caries have highlighted marked geographic variation in ECC prevalence across regions (33). Despite increased awareness of preventive strategies, ECC continues to impose a substantial global burden (34). The current study found that 60.9% of children aged 1–5 years had received dental treatment. While treatment receipt does not directly confirm ECC prevalence, this rate is consistent with the high burden of caries-related dental disease reported in this age group across the Gulf region, and underscores the need for preventive strategies targeting parents at an early stage.

*Reviewer comment: You could explicitly state: 'the results suggest that ECC knowledge in this population may be governed more by cultural and traditional factors than by formal education or income alone.'*

**Response:** This statement has been incorporated into the Discussion. The revised paragraph now explicitly states that ECC knowledge in this population may be governed more by cultural and traditional factors than by formal education or income alone. This framing was added at the end of the sociodemographic paragraph, which discusses higher knowledge scores among mothers, the association between educational attainment and ECC knowledge (19, 20), and the relatively low explanatory power of the regression model — indicating that cultural beliefs, access to oral health information, and health system engagement likely play a substantial additional role.

**Conclusion**

*Reviewer comment: Mention that the 'Well-Child Visit' (vaccination schedule) is the golden opportunity for this integration in the Saudi healthcare system.*

**Response:** This recommendation has been incorporated into the revised Conclusions section.

*Reviewer comment: Suggest a longitudinal study to see if knowledge eventually changes practice.*

**Response:** This suggestion has been added to the revised Conclusions and Future Directions section. We now recommend that future research employs longitudinal designs to determine whether improvements in parental knowledge, achieved through targeted educational interventions, translate into sustained changes in infant feeding and oral hygiene behaviors.

*Reviewer comment: Please add a practical implications section to demonstrate the clinical significance or the policy implications.*

**Response:** A dedicated ‘Clinical Implications’ subsection has been added at the end of the Discussion. “Dental health counseling should be integrated into routine pediatric primary care protocols, including the national well-child visit and vaccination schedule. Second, culturally adapted, Arabic-language educational materials targeting parents of young children should be developed and distributed through primary healthcare networks and digital platforms. Third, training programs for primary healthcare providers on guidance for oral health are needed. Finally, national oral health promotion policies should specifically address the early childhood period and integrate structured dental health education into the maternal and child health framework.”

We trust that the revisions made in response to the reviewers' and editors' constructive comments have substantially improved the manuscript. We remain committed to further clarification or revision should additional concerns arise.

We look forward to your favorable consideration of the revised manuscript.

Sincerely,

**Mohammed H. Alshamrani**, and co-authors

Department of Pediatric Dentistry and Orthodontics

King Saud University

Riyadh, Saudi Arabia

Email: malshamrani2@ksu.edu.sa
